# Association Between Polypharmacy and Self-Reported Hearing Disability: An Observational Study Using ATC Classification and HHIE-S-It Questionnaire

**DOI:** 10.3390/audiolres15050135

**Published:** 2025-10-10

**Authors:** Francesco Martines, Pietro Salvago, Gianluca Lavanco, Ginevra Malta, Fulvio Plescia

**Affiliations:** 1 Department of Biomedicine, Neuroscience and Advanced Diagnostics (BiND), Section of Audiology, University of Palermo, Via del Vespro, 129-90127 Palermo, Italy; francesco.martines@unipa.it; 2 Department of Health Promotion Sciences, Maternal and Child Care, Internal Medicine and Medical Specialties ‘Giuseppe D’Alessandro, University of Palermo, Via del Vespro, 133-90127 Palermo, Italy; gianluca.lavanco@unipa.it (G.L.); ginevra.malta@unipa.it (G.M.); fulvio.plescia@unipa.it (F.P.)

**Keywords:** polypharmacy, hearing loss, drugs-related side effects, ATC classification

## Abstract

Background: hearing loss represents, today, one of the most significant health problems affecting the world’s population. This clinical condition, particularly manifest in adulthood, can arise or be aggravated by both the presence of specific pathologies and by taking multiple classes of drugs at the same time. Methods: to understand this relationship, the present non-interventional observational study aimed to investigate the relationship between worsening hearing abilities in 1651 patients aged between 18 and 99 years. In particular, the thorough history of patients allowed us to evaluate the pathological profiles, pharmacological profiles, and therapeutic regimens adopted. This allowed us to evaluate its association with self-reported hearing loss, assessed through the administration of the HHIE-S-It questionnaire. Furthermore, given the presence of multimorbidity, the possible correlation between self-reported hearing loss and the specific classes of drugs, categorized using the Anatomical Therapeutic Classification (ATC) system, was evaluated. Results: the results highlighted how patients taking drugs, both in mono- and polytherapy regimens, had higher hearing deficits than patients not taking drugs. Furthermore, an apparent dose–response effect, in which the risk of moderate to severe impairment progressively increased with the number of drugs taken, was also observed. Different classes of drugs, particularly those used for the treatment of diseases of the cardiovascular system, as well as drugs for acid-related disorders, were significantly linked to an increased risk of perceived hearing impairment. On the contrary, agents belonging to the antidiabetic category have proven to be drugs capable of offering a potential protective effect. Conclusion: this study highlighted how both the number of drugs taken and some specific categories of drugs can contribute to perceived hearing impairment. While this evidence highlights the importance of integrating audiological evaluation into the management of patients in polypharmacy, the cross-sectional nature of the design precludes the inference of causality. This evidence still favors safer and more personalized therapeutic strategies.

## 1. Introduction

Hearing impairment represents a highly disabling clinical condition characterized by an alteration in the transmission of the acoustic signal along the auditory system from the external ear to the cortical areas responsible for sound processing [1]. According to the latest estimates by the World Health Organization (WHO), more than 5% of the world’s population, amounting to approximately 430 million people, including 34 million children, suffer from a clinically significant form of hearing loss. This condition requires appropriate prevention, treatment, and rehabilitation. Epidemiological projections suggest that by 2050, this figure could exceed 700 million cases, resulting in a substantial impact on quality of life and increasing pressure on global health systems [2,3,4,5].

In the Italian context, the most up-to-date data currently available [6] indicate an overall prevalence of hearing loss in the population of 12.1%, with significant differentiation among age groups: 13–45 (10%), 61–80 (25%), and over 80 (50%). In recent years, there has also been an increase in hearing deficits, not only in those over eighty but also in the age group between 46 and 60 years, suggesting an early trend of auditory degeneration linked to environmental and behavioral factors. In addition, several surveys have collected data based on self-assessed hearing impairment, a method that provides valuable though subjective insights into the perceived impact of auditory deficits [7,8,9].

One of the leading causes of hearing loss is presbycusis, a progressive neurosensory condition associated with the physiological aging of the auditory system [10]. However, the genesis of hearing loss is multifactorial: in addition to age-related degenerative processes, numerous elements can contribute to auditory impairment. Contributing factors include genetic predispositions, chronic noise exposure, otologic infections, inflammatory or autoimmune diseases, ototoxic drugs, polypharmacy, which can increase the risk of adverse drug reactions, and substance abuse [11,12,13,14,15,16,17].

Recent research suggests a clinical and pathophysiological interrelationship between hearing loss and several systemic chronic conditions. In particular, diabetic patients exhibit a higher incidence of hearing loss, likely mediated by microvascular and neuropathic mechanisms [18,19]. Similarly, psychosocial disorders such as depression and social isolation, which often coexist with hearing loss, can exacerbate the functional and cognitive impact [20,21,22]. Further associations have been highlighted for cardiovascular pathologies, chronic nephropathies, and alterations of the balance and vestibular apparatus. Cardiovascular dysfunctions, for example, can compromise cochlear perfusion, aggravating sensory damage [23]. Similarly, impaired kidney function has been associated with an increased risk of hearing loss, likely through shared mechanisms of metabolic toxicity and oxidative stress [24,25,26].

The management of these chronic conditions inevitably involves the use of pharmacological therapies, which may themselves influence auditory function. In patients living with chronic diseases, clinical management is based on the use of specific classes of drugs selected according to the individual pathological profile. Although many of these drugs are essential tools for controlling and slowing the progression of symptoms, they are not free from side effects. For example, some widely used molecules such as angiotensin-converting enzyme inhibitors (ACEi) and diuretics, frequently prescribed to manage different clinical conditions such as hypertension, heart failure, and certain kidney diseases, were associated with the occurrence of varying side effects [27,28]. Among these, some affect the auditory-vestibular apparatus, manifesting as dizziness, tinnitus, and impairment of hearing abilities [29,30]. On the contrary, other classes of drugs, such as antidiabetic agents, appear to play a protective role by reducing the underlying causes of hearing loss [31,32].

These observations raise important questions regarding how pharmacotherapy might contribute to or mitigate hearing impairment in patients with multimorbidity. To gain a deeper understanding of the relationship between hearing loss and chronic comorbidity, an insightful approach could involve analyzing the pharmacological profile of individual patients, correlating the pattern of drug intake in mono- or polytherapy with the level of auditory impairment. Different studies have shown that the presence of chronic diseases and the prevalence of subjects with multimorbidity lead doctors to prescribe multiple classes of drugs simultaneously, according to the various guidelines for each disease [33,34]. This results in a significant increase in the phenomenon of polytherapy, especially in the geriatric population, which is more prone to multiple chronic pathologies [35].

One of the consequences of polytherapy is the high rate of adverse reactions, mainly due to drug–drug interactions. The overall risk increases proportionally with the number of drugs taken and coexisting pathologies. In addition, the interaction between active ingredients can modify the pharmacodynamic and pharmacokinetic profile of each compound, making a potentially beneficial therapy harmful, especially in complex pharmacological regimens [36,37].

Although drug damage at the cochlear level has been extensively investigated in direct ototoxicity, with particular emphasis on receptor and neurosensory damage of the inner ear [38,39], there is still limited understanding of the role of drug therapy as an indirect mediator of hearing impairment in patients with chronic conditions. In particular, the interactions between the overall pharmacological load and auditory manifestations remain poorly explored, especially in clinical contexts of multimorbidity and polypharmacotherapy. Addressing this gap is crucial for developing more personalized and safer treatment strategies, particularly for vulnerable populations.

Based on the above, this observational work aimed to investigate the complex interplay between multimorbidity, polypharmacy, and hearing impairment. In particular, the objectives of this study were: (i) to characterize the pathological and pharmacological profile of patients with self-assessed hearing impairment; (ii) to explore the association between the therapeutic regimen (e.g., monotherapy vs. polytherapy and the number of medications taken) and the severity of self-assessed hearing impairment; (iii) to examine the correlation between specific classes of drugs, categorized by anatomical therapeutic chemical classification, and reported levels of hearing impairment, while also considering the coexistence of one or more chronic diseases. This approach is crucial and can help us to define safer and more personalized treatment strategies, reducing the risk of iatrogenic effects in particularly vulnerable patients.

## 2. Materials and Methods

### 2.1. Study Design and Participants

This non-interventional observational study was conducted at the Audiology Section of the University of Palermo between May 2021 and May 2025 and included the analysis of 1651 patients aged 18 to 99 years, comprising 905 males and 746 females (M/F ratio = 1.2); no significant differences in mean age were found between female and male patients. Participants were recruited among individuals presenting on the Audiological Section for a routine audiological check-up. Exclusion criteria were refusal to consent, severe cognitive impairment, and use of hearing aids for more than ten years, history of occupational or significant recreational noise exposure, history of head injury with reported auditory sequelae, and previous otologic surgery. All adult participants provided informed consent. Participants were volunteers and their data were handled with strict confidentiality. Most of our study population was between 50 and 89 years old (90.91%). Most of the patients were retired, while others held various types of jobs; some were unemployed, and some were homemakers. The identification of the type of work activity made it possible to subdivide the sample also according to the level of schooling.

All patients underwent a thorough anamnestic evaluation aimed at systematically and in detail collecting their individual medical history, detecting the presence of pathological conditions, both acute and chronic, the number of medications taken, and their classification. In addition, subjects included were evaluated through micro-otoscopy to rule out middle-ear pathologies and/or active infections.

Pure-tone audiometry (PTA) was performed by a trained audiologist with a Piano clinical audiometer (Inventis, Padua, Italy) in a soundproof audiometric room. Air conduction was measured without hearing aids using an on-ear TDH-49 headphones set to 250–8000 Hz; bone conduction was measured using a calibrated bone transducer at 250–4000 Hz.

To ensure the stability of middle ear function, tympanometry was performed using a Clarinet clinical tympanometer (Inventis, Padua, Italy) with a probe frequency of 226 Hz and an air pressure range of −400 to +200 daPa with automatic recording.

Although the primary outcome was self-reported hearing loss assessed with the HHIE-S questionnaire, all participants also underwent PTA and tympanometry as part of a standard clinical work-up. These tests were used to exclude conductive hearing loss from middle-ear pathology or active infection, thereby ensuring that the residual perceived hearing disability measured by the HHIE-S was predominantly sensorineural and pertinent to the study’s focus on multimorbidity and pharmacological load. PTA data additionally provided an objective clinical context for characterizing the study population. In parallel, the Italian version of the “Hearing Handicap Inventory—Screening Version” (HHIE-S-It) was administered to obtain a self-assessed measure of perceived hearing loss. Subsequently, three subgroups of patients were identified from the entire cohort analyzed: those who had not taken any medication in the 28 days prior to the visit (CTR = untreated group), those who had taken a single medication in the last 28 days prior to the visit (Pz1 = single-drug group); while the third group included patients treated with multiple (two or more drugs) for at least 28 days prior to the visit (Pz+ = polypharmacy group) (Table 1). This distinction made it possible to conduct a comparative analysis aimed at exploring the possible association between the number of drugs taken and the subjective perception of auditory impairment.

In addition, within the Pz1 and Pz+ groups, further stratification was carried out based on the Anatomical Therapeutic Chemical (ATC) Classification of the active substance taken. This stratification enabled the investigation of a possible correlation between specific pharmacological classes and the degree of self-reported hearing loss, aiming to identify potential hearing deficits associated with the intake of a particular therapeutic category.

All participants were informed about the purpose of the study and subsequently asked to sign an informed consent form, which allowed for the processing of their data in a confidential manner. Filling out the questionnaire used (described in the following paragraph) was on a voluntary basis.

All procedures have been conducted in accordance with the ethical principles outlined in the Declaration of Helsinki, and data have been managed in compliance with Italian law for the protection of personal data (Decree No. 196, January 2023).

### 2.2. Classification and Quantification of Drugs Taken According to the ATC System

Evaluation of drug use was conducted using a set of structured questions specifically designed for this study, adapted from the Trimbos/iMTA Questionnaire for Costs Associated with Psychiatric Illness [40]. Participants were asked if they had taken any drugs (e.g., antidyslipidemic drugs, acetylsalicylic acid) in the last 28 days. It was also specified that drugs taken during hospital admissions should not be included (to focus on chronic outpatient medication use) and, for female patients, oral contraceptives should be excluded (as they are generally not considered chronic disease management medications in the context of multimorbidity). If yes, patients provided the name of the drug or drugs, the frequency of daily intake, and the number of days that the drug was taken in the last 28 days.

The categorization of reported drugs was performed in a comprehensive way using the “ATC” System authorized by the World Health Organization in 2003. The ATC system is designed to classify drugs into distinct types based on their anatomical and therapeutic properties. The classification included drugs from all major ATC level one groups identified in the study population [41].

### 2.3. Self-Reported Hearing Impairment

The assessment of hearing impairment was conducted through the administration of the Italian version of the “Hearing Handicap Inventory—Screening Version” (HHIE-S-It), a valuable tool to assess how an individual perceives the social and emotional effects of hearing loss [42]. The examination conducted through this instrument enables the evaluation of self-mediated hearing impairment and serves as a good starting point for screening hearing loss.

This tool consists of a 10-question questionnaire, which is administered on paper and pencil. It consists of two subscales, each with five items, that assess the self-perceived impact of hearing loss in the emotional (E) and socio-situational (S) domains, respectively. Each question has three potential answers: “Yes” gets 4 points, “Sometimes” gets 2 points, and “No” gets 0. The scoring system classifies hearing impairment based on the total score (ranging from 0 to 40 points) as follows: no hearing impairment (0–8), moderate hearing impairment (10–24), and significant hearing impairment (26–40).

## 3. Statistical Analysis

Data analysis was conducted using the open-source software Jamovi 2.6.26 (Sydney, NSW, Australia). Before proceeding with the statistical analysis, all the collected data have been organized to allow for the different analyses planned. To analyze the correlation between a specific ATC class and the self-administered hearing impairment score, a binomial logistic regression was carried out. In this model, the presence or absence of self-reported hearing impairment (yes/no) was considered as a dependent variable, while ATC classes represented the main independent variables. Age, sex, and level of education were included as covariates.

In order to understand whether there was an association between the number of drugs taken and the risk of self-reported hearing impairment, a multinomial logistic regression was conducted. In this case, the dependent variable was represented by three levels of hearing impairment (absence, moderate, significant), while the number of drugs taken was the primary independent variable; age, sex, and level of education were considered covariates.

Finally, to understand whether there were differences in the degree of hearing impairment between patients who had taken drugs in the 28 days prior to the visit and those who had not taken any drug therapy, an ordinal logistic regression was conducted. The dependent variable was the categorized self-assessed hearing impairment (no, moderate, severe), while the independent variables included medication (yes/no), sex, and education level; age was considered a covariate.

## 4. Results

### 4.1. Assessment of Self-Reported Hearing Impairment

**Figure 1:**
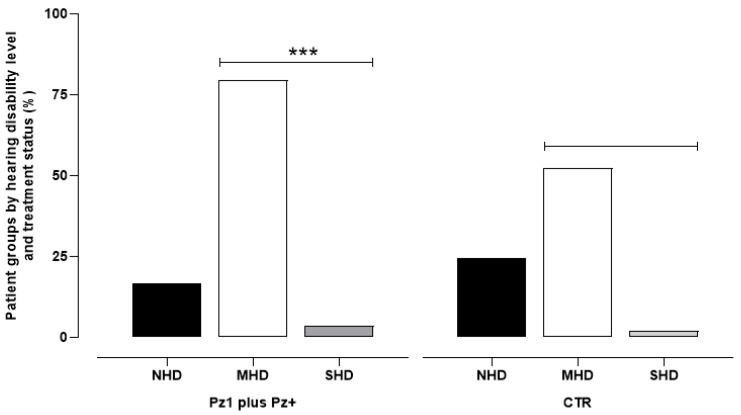
The data represent the percentage of 1224 patients undergoing pharmacological therapy (Pz1 = 363 plus Pz+ = 861) and 437 control patients (CTR) who had not taken any medication for at least 28 days prior to their visit. The categories include: No Hearing Disability (NHD), Moderate Hearing Disability (MHD), and Severe Hearing Disability (SHD). *** *p* < 0.001 vs. respective CTR.

The analysis of the HHIE-S-It questionnaires showed that, among the 1224 patients under drug therapy, 16.83% had no hearing impairment, while the remaining 83.17% had moderate (79.58%) or severe (3.59%) self-reported hearing impairment. Among the patients who had not taken any medication for at least 28 days prior to their visit, 31% did not have self-reported hearing impairment, 66.5% reported moderate disability, and the remaining 2.5% reported a severe disability (Figure 1).

In order to understand if there were differences in self-reported hearing impairment between subjects on and off treatment, an ordinal logistic regression analysis was conducted. The results of the statistical analysis showed that medication use is associated with a significantly higher probability (OR = 1.749; 95% CI: 1.11–2.85; *p* < 0.001) of hearing impairment compared to patients not undergoing pharmacological treatment. In addition, it was found that as patients grow older, the probability of developing hearing loss is statistically significant (OR = 1.026; 95% CI: 1.015–1.04; *p* < 0.001), as well as patients with a lower level of education (average license) are more likely to develop hearing impairment (OR = 1.799; 95% CI: 1.036–3.11; *p* < 0.001) than those with a higher education qualification.

### 4.2. Self-Assessed Hearing Impairment Related to a Specific ATC Category

To understand whether there was a correlation between the intake of drugs belonging to a specific ATC class and the presence of self-reported hearing impairment, a statistical analysis was conducted using a binomial logistic regression model. The analysis included only patients who reported using at least one drug and evaluated the association between the pharmacological class of the active pharmaceutical ingredients taken and the presence or absence of self-mediated hearing impairment (Table 2). The results showed a statistically significant association between hearing impairment and certain major drug classes. In particular, medication for the treatment of disorders associated with gastric acid secretion was found to be associated with a significant increase in reporting hearing impairment (OR = 3.336; 95% CI: 1.732–6.53; *p* < 0.001) compared to patients who were not taking drugs of this class. In contrast, patients receiving antidiabetic therapy had a significantly reduced likelihood of hearing impairment (OR = 0.734; 95% CI: 0.542–0.994; *p* < 0.046), suggesting a potential protective effect from this drug category. The use of antithrombotics was also associated with an increased risk of self-reported hearing impairment (OR = 3.540; 95% CI: 2.280–5.50; *p* < 0.001) compared to those who did not use these drugs.

The analysis also showed that pharmacological therapy with drugs used in the modulation of the cardiovascular system was significantly associated with a higher probability of hearing impairment. Specifically, the classes involved included: Antihypertensives (OR = 6.005; 95% CI: 1.449–24.89; *p* < 0.001); Diuretics (OR = 4.164; 95% CI: 2.32–7.47; *p* < 0.001); Beta blocking agents (OR = 4.135; 95% CI: 2.645–6.46; *p* < 0.001); Calcium channel blockers (OR = 3.287; 95% CI: 1.951–5.54; *p* < 0.001); Agents acting on the renin-angiotensin system (OR = 4.084; 95% CI: 2.843–5.86; *p* < 0.001) and Lipid modifying agents (OR = 3.425; 95% CI: 2.18–5.288; *p* < 0.001). No statistically significant differences with hearing impairment were found for all other ATC categories analyzed, including those with insufficient sample size for robust statistical analysis.

### 4.3. Assessing Self-Reported Hearing Impairment in Relation to the Number of Medications Used

The analysis of data relating to the evaluation of self-medicating hearing impairment has highlighted a significant association between the number of drugs taken and the risk of experiencing an impairment in auditory abilities. Statistical analysis conducted through a multinomial logistic regression showed that the increase in the number of drugs taken was closely associated with a progressive and statistically significant increase in the risk of developing an auditory disability, both moderate and high.

When we looked at patients who were assessed as having moderate HHIE-S-It deficits, it was found that the risk increased by more than three times with two drugs (OR = 3.20; 95% CI: 2.11–4.85; *p* < 0.001] and reached very high levels with four or more drugs. Similarly, for patients with severe hearing impairment, association with the number of drugs taken was marked, with OR increasing dramatically for three drugs (OR = 4.20; 95% CI: 0.98–18.05; *p* = 0.053) and significantly higher for four (OR = 122.6; 95% CI: 27.78–540.58; *p* < 0.001) e five drugs (OR = 112.76; 95% CI: 25.19–504.70; *p* < 0.001).

Among the other factors, age emerged as a significant predictor of severe hearing loss, with additional years associated with a higher risk (OR = 1.039 per year; 95% CI: 1.002–1.078; *p* = 0.038). Conversely, age showed a protective effect against moderate hearing loss (OR = 0.982; 95% CI: 0.968–0.996; *p* = 0.012). Sex and education did not significantly predict hearing loss in any of the comparisons (Table 3).

It is essential to note that, although the OR observed for patients taking six or more drugs, its interpretation must be taken with caution due to the scarcity of data on the number of patients in these higher drug count categories. These extremely large OR, particularly those with single-point confidence intervals, often indicate issues of quasi-complete or complete separations in the logistic regression model, where there are very few or no observations in certain outcome categories at these extreme levels of drug use. While suggesting a strong association, these specific values are likely unstable and less reliable as precise estimates. This condition may affect the accuracy of the estimate made.

## 5. Discussion

This non-interventional observational study investigated the possible association between medication use and the presence of self-reported hearing impairment in a cohort of adult patients with heterogeneous clinical conditions. Beyond simply identifying associations, this work explores the qualitative and quantitative impact of pharmacological load on patient-perceived hearing function. The findings suggest that both the chronic use of medication and the total number of drugs prescribed to manage multiple chronic diseases may represent potential predisposing factors for hearing impairment. Furthermore, some active substances belonging to different therapeutic classes appear particularly prone to causing auditory deterioration, outlining a clinical scenario, especially considering the increasing prevalence of multimorbidity and the consequent widespread use of chronic polypharmacy, particularly among older adults [43].

The observation that patients undergoing pharmacological treatment are more likely to report symptoms of hearing impairment than those not receiving drug therapy is consistent with the literature, which highlights the complex interplay between aging, disease chronicity, and prolonged pharmacological exposure [44]. Aging and chronic conditions, which often require continuous and complex therapeutic regimens, are frequently associated with a progressive decline in auditory function [9]. Presbycusis, one of the most common forms of hearing loss in older adults, is a multifactorial degenerative process involving neurosensory deterioration, alterations in cochlear microcirculation, and metabolic imbalances at the cochlear level [45,46].

Notably, an observed association exists between the risk of hearing impairment, both moderate and severe, and the number of medications taken. While our findings for very high numbers of drugs (e.g., six or more) resulted in exceptionally large odds ratios, likely indicative of quasi-complete separation due to limited observations in these categories, the overall trend unequivocally demonstrates that increased medication count correlates with a progressively higher risk of self-reported hearing impairment. This correlation reinforces concerns previously documented in the literature regarding the risks associated with the concurrent use of multiple pharmacological agents. While polypharmacy is often necessary for individuals suffering from multiple chronic illnesses, it is also associated with an increased risk of several adverse outcomes, including frailty, functional decline, hospitalization, morbidity, and mortality [47,48]. Additionally, the use of multiple medications increases the likelihood of drug-related adverse events, drug–drug interactions, and a general deterioration in the patient’s overall functional status [49].

The analysis conducted to evaluate the relationship between self-reported hearing impairment and specific categories of ATC-classified drugs revealed several statistically significant associations. In particular, multiple classes of cardiovascular medications, including antihypertensives, diuretics, beta-blockers, calcium channel blockers, agents acting on the renin-angiotensin system, and lipid-lowering drugs, were strongly associated with hearing impairment, consistent with evidence that cardiovascular risk factors, such as hypertension, dyslipidemia, systemic arteriosclerosis, and diabetes, contribute to cochlear dysfunction via microcirculatory changes, oxidative stress, and chronic inflammation [50,51,52]. Many of these drugs also have direct ototoxic and vestibular effects, such as dizziness and postural instability, as documented for loop diuretics such as furosemide and for ACE inhibitors like enalapril [39,53,54,55,56,57].

Non-selective beta-blockers, such as carvedilol, appear more strongly associated with hearing loss than selective beta-blockers, such as metoprolol and bisoprolol [58], possibly due to alpha-1-mediated reductions in cochlear perfusion rather than beta-receptor blockade per se [59].

Statistically significant associations were also identified between antithrombotic drugs and increased self-reported hearing impairment. Antithrombotic agents, including acetylsalicylic acid, are known to induce tinnitus and reversible hearing loss by disrupting cochlear electrolyte balance, impairing outer hair cell function through prestin inhibition, reducing blood flow, and increasing oxidative stress, as well as potentially modulating neurotransmitter activity [60,61,62,63,64,65,66,67].

Higher HHIE-S-It scores were also observed among users of drugs for gastric disorders, predominantly proton pump inhibitors (PPIs). Long-term use has been linked to sensorineural hearing loss and tinnitus through multiple pathways, including endothelial dysfunction with cochlear ischemia, which is mediated by reduced endothelial nitric oxide synthase (eNOS) activity and increased levels of asymmetric dimethylarginine (ADMA) [68,69,70,71,72,73]. Impaired absorption of micronutrients such as vitamin B12 [74,75,76], inhibition of a cochlear isoform of H+/K+-ATPase crucial for endolymph homeostasis [77], and increased susceptibility to upper respiratory tract infections such as otitis media [78,79]. Experimental data also suggest neurodegenerative effects resulting from central accumulation of β-amyloid [80,81].

A notable finding emerged from the analysis of data on patients undergoing drug therapy for diabetes. Although diabetes is a well-established risk factor for hearing loss, likely mediated by microvascular and neuropathic mechanisms [82], subjects on antidiabetic therapy reported scores on the HHIES-S-It questionnaire indicating a lower subjective perception of hearing impairment. Based on this evidence, the pharmacological profile of the participants was analyzed to identify the therapeutic class that was predominantly used. The data showed that approximately 78% of patients were taking drugs belonging to the class of biguanides, 7% GLP-1 agonists, and the remaining 15% were in pharmacological therapy with insulin, alone or in combination, for the treatment of type 1 diabetes. Considering that most patients were taking metformin, this drug may exert a protective effect against the pathophysiological mechanisms contributing to hearing loss, although this hypothesis requires further investigation in clinical and experimental settings. Some scientific research has shown that the use of metformin, in addition to its known hypoglycemic effects, may have a protective effect on hearing through different molecular mechanisms. In particular, through the activation of AMP-activated protein kinase (AMPK), a kinase with a central role in cellular energy homeostasis, metformin may reduce vascular inflammation and protect the endothelium through the activation of nitric oxide synthesis (nitric oxide synthase) [83,84]. Furthermore, in vitro studies have demonstrated that metformin can protect cochlear hair cells from death induced by ototoxic agents by reducing the production of reactive oxygen species (ROS) [84]. The AMPK pathway may also trigger cellular signaling cascades that protect the inner ear from damage induced by prolonged or excessive acoustic stimulation [84].

These results suggest that perceived hearing impairment may reflect a broader decline in physiological resilience associated with cumulative drug exposure, particularly in the context of polypharmacy. It is important to note that although a direct causal link between polytherapy and hearing loss has not been demonstrated, the increase in drug load may contribute to increased physiological and systemic vulnerability, predisposing the individual to adverse effects on the auditory apparatus.

Reverse causality should also be taken into account. Sicker individuals overall will consume more medications and possibly have more severe hearing difficulties anyway, regardless of the direct effects of the drugs. It is worth noting that the relationship between polypharmacy and self-reported hearing impairment may be partly attributed to a heightened overall systemic susceptibility or reduced physiological resistance to multimorbidity and long-term illness. While our observational study cannot establish a cause-and-effect relationship, further studies involving objective audiometric testing, as well as patient symptom surveys, will be essential to discern the effect of medication from the effects of age or comorbid illnesses.

Notably, the HHIE-S-It questionnaire assesses the psychosocial impact of hearing loss on the individual, rather than quantifying auditory thresholds through objective audiometry. This study relied on the subjective perception of hearing difficulty. While not an audiometric assessment, the HHIE-S-It captures the psychosocial impact of hearing loss, which is increasingly recognized as clinically relevant. Further studies combining objective audiological assessments with patient-reported outcomes are needed to elucidate the potential otoprotective effects of specific antidiabetic drugs and disentangle them from broader age- or disease-related vulnerabilities.

## 6. Conclusions

Data collected contributes to an understanding of the relationship between drug load and perceived hearing impairment in the adult population. In a clinical context increasingly characterized by population aging and multimorbidity, our results suggest that pharmacological exposure, both in quantitative and qualitative terms, could represent an additional factor affecting auditory function. Therefore, careful consideration is warranted in medical practice. The identification of specific classes of drugs associated with an increased risk of hearing loss, together with the potential protective effects of some antidiabetics, lays the groundwork for future research aimed at elucidating the biological mechanisms underlying these associations and developing more personalized therapeutic pathways. Considering the possible effects of pharmacological therapies on hearing abilities more carefully, both in the selection and monitoring phases, is paramount for safeguarding patients’ quality of life, particularly those with complex clinical conditions.

Future studies should combine subjective questionnaires with audiometric data to confirm these associations and identify protective or harmful agents.

## 7. Limitations of the Study

Like other observational studies, this study has several important limitations that must be recognized. The first concerns the use of a subjective assessment tool that measures an individual’s perception of hearing impairment, rather than including hearing thresholds data. Although this approach presents a limitation in terms of clinical quantifying sensory impairment, it aligns with our primary goal of exploring the functional and psychosocial impact of hearing loss as experienced and reported by patients, consistent with a person-centered care model. Therefore, while offering a valuable insight into perceived disability, these findings should be interpreted cautiously regarding direct audiometric correlation. In addition, the study’s cross-sectional design did not allow for a causal link to be established between drug use and hearing impairment; however, it did identify some clinically relevant trends. Finally, it is not possible to completely exclude the possibility of underestimation or overestimation in the self-reporting of drugs taken, despite the meticulous ATC adopted. Despite these limitations, the results obtained provide some clinical insight and represent a good starting point for understanding the possible impact that an excessive pharmacological load can have on patients’ auditory health.

## Figures and Tables

**Table 1:** Characteristics of the population studied. For the number and total population, percentages were calculated based on the total number of patients (*n* = 1651). For Pz1 and Pz+, percentages were calculated within their respective subgroups (n = 363 for Pz1 and n = 861 for PZ+). CTR: patients who had not taken any medication in the 28 days prior to the visit; Pz1: patients who had taken at least one medication in the 28 days preceding the visit; Pz+: patients taking multiple medications for at least 28 days prior to the visit; n: total number of patients; % is a percentage.

		Total Population *n* = 1651 R Plus Pz1 Plus Pz+)		Use of at Least One Drug in the Last 28 Days (*n* = 363) Pz1	Use of More than One Drug for at Least 28 Days (*n* = 861) Pz+
***Sociodemographic descriptives***		n	%	%	%
***Gender***					
	Male	905	54.82	50.96	57.03
	Female	746	45.18	20.67	42.97
***Age***					
	18–29	10	0.61	66.67	33.33
	30–39	20	1.21	100.00	0.00
	40–49	87	5.27	37.04	62.96
	50–59	301	18.23	43.50	56.50
	60–69	433	26.23	32.98	67.02
	70–79	485	29.38	22.77	77.23
	80–89	282	17.08	21.84	78.16
	90–99	33	2.00	31.25	68.75
***Level of Schooling***					
	Low	378	22.90	33.21	66.79
	Mid	611	37.01	28.19	71.81
	High	320	19.38	32.22	67.78
	Unknown	342	20.71	25.97	74.03

**Table 2:** Association between the presence of self-diagnosed hearing impairment and the different ATC classes of drugs taken. Adjusted ORs are presented. The analyses included age, sex, and level of education as covariates. The results have been adjusted for these variables to control for potential confounding effects. ORs in groups with <10 subjects should be interpreted with caution due to limited statistical power.

ATC Use Category			Subjects	Adjusted Models		
Main Group	Sub-Group		*n*°	OR	95% CI	*p* Value
**A**		**Alimentary tract and metabolism**				
	A02	Drugs for acid-related disorders	157	3.336	1.732–6.53	**0.001**
	A06	Drugs for constipation	8	-	-	-
	A07	Antidiarrheals, intestinal anti-inflammatory/anti-infective agents	8	-	-	-
	A10	Drugs used in diabetes	479	0.734	0.542–0.994	**0.046**
	A11	Vitamins	10	-	-	-
**B**		**Blood and blood-forming organs**				
	B01	Antithrombotic agents	367	3.540	2.280–5.50	**0.001**
	B03	Antianemic preparations	4	-	-	-
**C**		**Cardiovascular System**				
	C01	Cardiac therapy	42	4.082	0.974–17.11	0.054
	C02	Antihypertensives	62	6.005	1.449–24.89	**0.013**
	C03	Diuretics	234	4.164	2.321–7.47	**0.001**
	C07	Beta blocking agents	386	4.135	2.645–6.46	**0.001**
	C08	Calcium channel blockers	253	3.287	1.951–5.54	**0.001**
	C09	Agents acting on the renin-angiotensin system	556	4.084	2.843–5.86	**0.001**
	C10	Lipid-modifying agents	362	3.425	2.218–5.288	**0.001**
**G**		**Genito urinary system and sex hormones**				
	G04	Urologicals	70	1.046	0.761–1.438	0.783
**H**		**Systemic hormonal preparations, excl. Sex hormones and insulins**				
	H03	Thyroid therapy	80	0.8701	0.6359–1.191	0.358
	H05	Calcium homeostasis	2	-	-	-
**L**		**Antineoplastic products, insecticides and repellents**				
	L01	Antineoplastic agents	6	-	-	-
	L02	Endocrine therapy	8	0.630	0.125–3.18	0.575
**M**		**Musculoskeletal system**				
	M01	Anti-inflammatory and antirheumatic products	6	0.358	0.0640–2.00	0.242
	M04	Antigout preparations	20	0.460	0.173–1.22	0.119
	M05	Drugs for the treatment of bone diseases	6	-	-	-
**N**		**Nervous system**				
	N02	Analgesics	2	-	-	-
	N03	Antiepileptics	14	1.213	0.267–5.51	0.802
	N04	Anti-Parkinson drugs	16	1.205	0.265–5.48	0.809
	N05	Psycholeptics	19	0.739	0.241–2.26	0.596
	N06	Psychoanaleptics	20	0.451	0.167–1.22	0.117
**R**		**Respiratory system**				
	R03	Drugs for obstructive airway diseases	40	1.126	0.464–2.273	0.793
	R05	Cough and cold preparations	2	-	-	-
	R06	Antihistamines for systemic use	6	0.399	0.0719–2.21	0.293
**S**		**Sensory organs**				
	S01	Ophthalmologicals	12	1.040	0.225–4.80	0.960

**Table 3:** Data obtained from the multinomial logistic regression analysis. HHIE-S-It: self-reported hearing impairment; 0 = no hearing impairment; 1= moderate hearing impairment; 2 = significant hearing impairment. The estimates were based on the category of moderate or severe hearing impairment compared to the category of no hearing impairment. Odds Ratios (ORs) for the categories of six or more drugs are considerably overestimated. This instability is a result of the limited number of patients in such extreme subgroups (n < 20 for each category) and potential quasi-complete separation, leading to wide confidence intervals. These values primarily serve to indicate the direction of rising risk and must not be over-interpreted for their precise magnitude.

HHIE-S-It	Variable	OR	95% CI	*p* Value
1-0	Intercept	5.269	2.077–13.36	0.001
	Gender	0.921	0.664–1.279	0.626
	Age	0.982	0.968–0.996	0.012
	Level of Schooling	1.051	0.893–1.237	0.548
	Number of drugs used			
	2-1	3.196	2.107–4.848	0.001
	3-1	6.299	3.998–9.926	0.001
	4-1	23.478	8.486–64.956	0.001
	5-1	12.111	4.289–34.200	0.001
	6-1	5.12 × 10^7^	2.49 × 10^7^–1.05 ×10^8^	0.001
	7-1	622,074	622,074–622,074	0.001
	8-1	1.14 × 10^6^	1.14 × 10^6^–1.14 × 10^6^	0.001
	9-1	1.09 × 10^6^	1.09 × 10^6^–1.09 × 10^6^	0.001
2-0	Intercept	0.004	2.85 × 10^−4^–0.0626	0.001
	Gender	0.804	0.395–1.638	0.548
	Age	1.039	1.002–1.078	0.038
	Level of Schooling	0.748	0.523–1.059	0.102
	Number of drugs used			
	2-1	6.45 × 10^−8^	6.45 × 10^−8^–6.45 × 10^−8^	0.001
	3-1	4.204	0.979–18.054	0.053
	4-1	122.55	27.783–540.58	0.001
	5-1	112.75	25.191–540.70	0.001
	6-1	1.26 × 10^4^	6.11 × 10^7^–2.58 × 10^8^	0.001
	7-1	1.276	1.276–1.276	0.001
	8-1	0.593	0.594–0.5936	0.001
	9-1	0.526	0.526–0.526	0.001

## Data Availability

The datasets are not publicly available. Anonymized data may be provided upon request by Fulvio Plescia.
